# Assessing cataract patients at the treatment centre and allocating the right surgeon

**Published:** 2025-11-22

**Authors:** Andrew Blaikie, Lila Raj Puri, Louis Oteng-Gyimah

**Affiliations:** 1 Senior Lecturer, University of St Andrews, Scotland, UK.; 2 Medical Advisor, Asia: The Fred Hollows Foundation, London, UK.; 3 Medical Director/Ophthalmologist in charge: Anglican Eye Clinic, Jachie, Ashanti Region, Ghana.

**A well-structured preoperative process minimises intraoperative surprises and improves surgical outcomes.**

**Figure:**
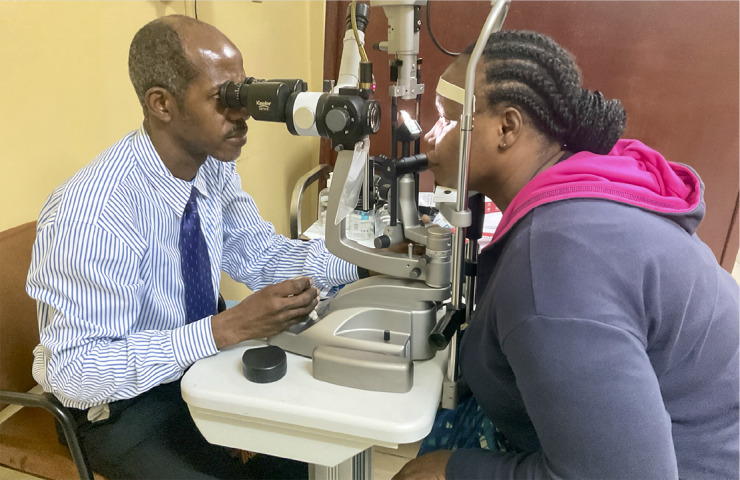
Preoperative evaluation of a patient before cataract surgery. SIERRA LEONE

© UNISA KAMARA CC BY-NC-SA 4.0

Patients referred from community or primary eye care centres for cataract surgery require systematic evaluation before surgery. The aim is to confirm the diagnosis, rule out contra-indications, grade the surgical complexity, and ensure the patient is ready for surgery. A well-structured preoperative process minimises intraoperative surprises and improves surgical outcomes.

A cataract diagnosis should not rely solely on lens opacity, but vision must correlate with cataract density and no other pathology. It is helpful to identify patients unlikely to benefit fully from surgery due to coexisting disease of the front or back of the eye.

## Assessment: the steps

**Record** each patient's name and phone number and the details of the person or centre responsible for referring them. It is helpful to let the referral centre or person know the names of everyone who attended, so they can follow up with those who haven't. This improves continuity of care, and it is also an opportunity to give feedback about the accuracy of each referral.

**Take a careful history**, focusing on the impact of vision loss on activities of daily living, previous ocular surgery or trauma, the history of systemic diseases (diabetes, hypertension, bleeding disorder, etc.) and current medications.

**Assess visual acuity** for distance and near, both unaided and with a pinhole, for both eyes. This establishes baseline vision and confirms the impact of cataract on visual function. Carry out refraction if vision improves with pinhole or when media clarity allows.

Assess the **intraocular pressure**.

Before dilation, **check pupil response** (constriction to a bright light in a dim room).

Examine the **anterior segment** using a slit lamp to evaluate the eyelids, conjunctiva, and cornea (to check clarity and identify any disorders), the anterior chamber depth, and the cataract density and type.

**Figure 1:**
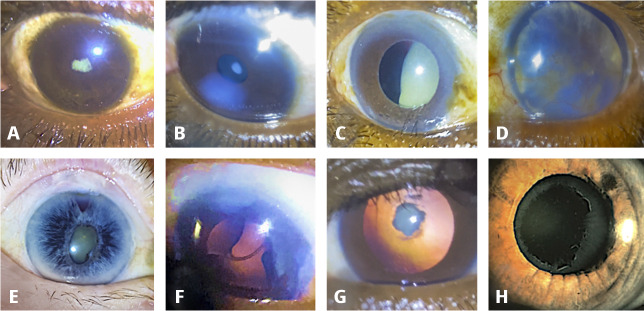
Anterior segment comorbidities and/or risk factors for challenging cataract surgery: **A.** Pupil with synechia **B.** Hazy cornea **C.** Phacodenesis **D.** Dislocated lens in anterior chamber **E.** Previous trabeculectomy **F.** Previous complicated cataract surgery **G.** Posterior polar cataract **H.** Pseudoexfoliation.

Check for **comorbidities and/or risk factors**; for example: a pupil with synechia, a hazy cornea, phacodenesis (movement of the lens due to lack of zonular support), a dislocated lens in the anterior chamber, a previous trabeculectomy, previous complicated cataract surgery, posterior polar cataract, and pseudo-exfoliation (Figure 1 A–H).

© LILA RAJ PURI CC BY-NC-SA 4.0

**Figure 2:**
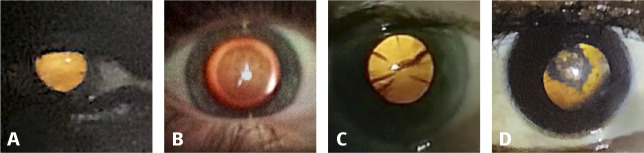
Dilated fundal reflex examination to check for the presence and type of cataract. **A.** Clear media – no cataract. **B.** Nuclear cataract. **C.** Cortical cataract. **D.** Posterior subcapsular cataract.

© ANDREW BLAIKIE CC BY-NC-SA 4.0

**Figure 3:**
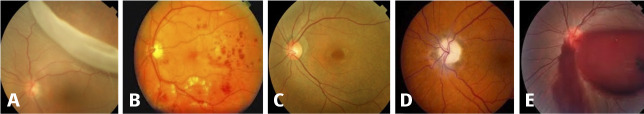
Posterior segment comorbidities and/or risk factors for challenging cataract surgery: **A.** Retinal detachment, **B.** Diabetic retinopathy, **C.** Macular hole, **D.** Optic atrophy, **E.** Vitreous haemorrhage.

**After dilation**, record the size of the pupil and examine the **fundal reflex** for the presence and type of cataract (Figure 3).

© LILA RAJ PURI CC BY-NC-SA 4.0

Check for any **comorbidities of the posterior segment**, such as retinal detachment, diabetic retinopathy, macular hole, optic atrophy, or vitreous haemorrhage (Figure 3 A–E).

**Figure 4:**
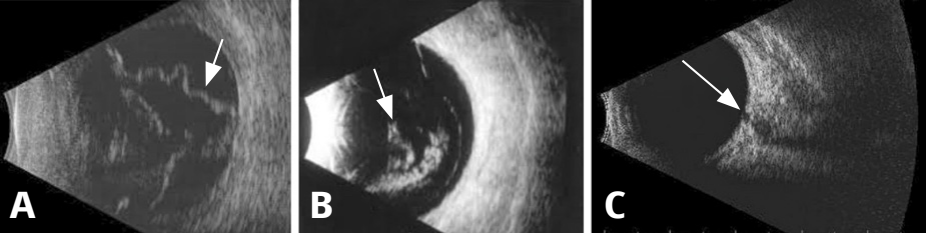
B-scan showing posterior segment pathology: **A.** Retinal detachment **B.** Vitreous haemorrhage **C.** Optic disc cupping.

If the **fundus view is obscured**, especially if the initial pupil constriction to bright light was abnormal, perform a **B-scan** to rule out disease at the back of the eye (Figure 4 A–C).

© LILA RAJ PURI CC BY-NC-SA 4.0

## Making a decision: operate now, defer, or offer alternative management

Operating on patients that have other active disease or causes of visual impairment can lead to poor outcomes. This can potentially serve as bad publicity in the community and discourage other people with cataract, who could benefit from surgery, from attending.

Deciding whether a patient with cataract should undergo surgery immediately, be advised to defer surgery, or receive alternative management, is a critical step in cataract care. This decision must balance visual needs, ocular and systemic risk factors, patient expectations, and service capacity. While cataract surgery remains the definitive treatment for visually significant lens opacity, not every patient referred for surgery is ready or suitable at that moment. The evaluation process should therefore aim to identify those who will benefit most from timely intervention, those who require stabilisation or further investigation/treatment before surgery, and those for whom non-surgical or low-vision care is more appropriate.

Patient preference, general health, and the presence of other ocular diseases should also be considered before making the final decision.

We suggest the following criteria.

### 1. Operate now

Operate now if:

- The vision loss is due to cataract rather than another eye condition
- The cataract is significantly affecting the person's quality of life, such as difficulty reading, driving, or performing routine tasks
- There are no contraindications, such as active infection, uncontrolled systemic illness, or ocular inflammation
- The patient is willing to accept surgery, understands the risks and benefits, and gives informed consent.

If the decision is to go ahead with cataract surgery, the next step is biometry, including axial length and keratometry, to calculate the intraocular lens (IOL) power to ensure postoperative refractive predictability and patient satisfaction.

Carry out a systemic assessment, checking blood pressure, blood sugar, and overall suitability for local anaesthesia to minimise the risk of complications during surgery. Offer advice on blood sugar management if levels are high.

Discuss as realistic visual prognosis with the patient, especially if comorbidities present, and the choice of IOL (see bit.ly/43RJh8m). Talk about the anaesthesia plan, the need for someone to accompany them, the direct and indirect cost considerations, and the postoperative follow-up, medication and schedule.

### 2. Defer surgery

Defer surgery if:

- There are other active eye conditions. These need to be treated first, e.g. conjunctivitis, corneal infection, active inflammation, advanced diabetic retinopathy, or another posterior segment disease causing vision impairment
- The cataract is immature, and vision can be adequately corrected with glasses. Tell the patient to return if they start to struggle with activities of daily living
- The patient suffers from uncontrolled systemic illness (e.g., severe hypertension, hyperglycaemia, cardiac instability). These need to be controlled first.

### 3. Offer alternative management

Offer low vision and rehabilitation support to patients with advanced glaucoma (end-stage glaucoma), visually significant corneal scarring, or advanced age-related macular degeneration. Take time to explain to them why cataract surgery will not improve their vision.

## Case complexity grading: who should operate?

Surgical complications, due to inexperienced surgeons operating on eyes with complex cataracts, can also result in disappointing patient outcomes. This can potentially also serve as bad publicity. Grading the difficulty of the operation based on the nature of the eye and patient, and identifying an appropriate surgeon based on seniority/experience to operate, is therefore important. Case allocation optimises outcomes, builds progressive surgical competency, and reduces complication rates. The following can aid as a guide in this process.

**Junior trainee, with supervision.** Catarat with good dilation, emmetropic eye, no pseudoexfoliation or Fuch's dystrophy, easy access to the eye, with a healthy fellow eye, in a cooperative patient with minimal/no comorbidities.

**Senior trainee, with indirect supervision.** Mature cataract, small pupil, pseudoexfoliation with minimal phacodonesis, mild Fuch's dystrophy, relatively cooperative patient with acceptable access to eye.

**Experienced senior consultant.** Small pupil, hypermature/brunescent lens, pseudoexfoliation with obvious phacodenesis or subluxation, history of eye surgery or trauma, corneal scar obscuring view, high myopia or hypermetropia, only one good eye, or a very deep-set eye with a prominent brow.

**Anterior vitrectomy**

Every cataract centre must ideally be equipped with an anterior vitrectomy system, and every cataract surgeon, irrespective of their speciality, must know how to do a good anterior vitrectomy. This reduces the incidence of retinal detachment dramatically. In addition, if there is vitreous haemorrhage or obvious coexistent proliferative diabetic retinopathy, then combined cataract extraction, vitrectomy, and laser in a tertiary centre is preferred.
